# Supplementary figures and images for: Whole-Genome Characterisation of ESBL-Producing E. coli Isolated from Drinking Water and Dog Faeces from Rural Andean Households in Peru
Source: Antibiotics (Basel). 2022 May 20;11(5):692. doi: 10.3390/antibiotics11050692 (PMC9137468; doi:10.3390/antibiotics11050692)

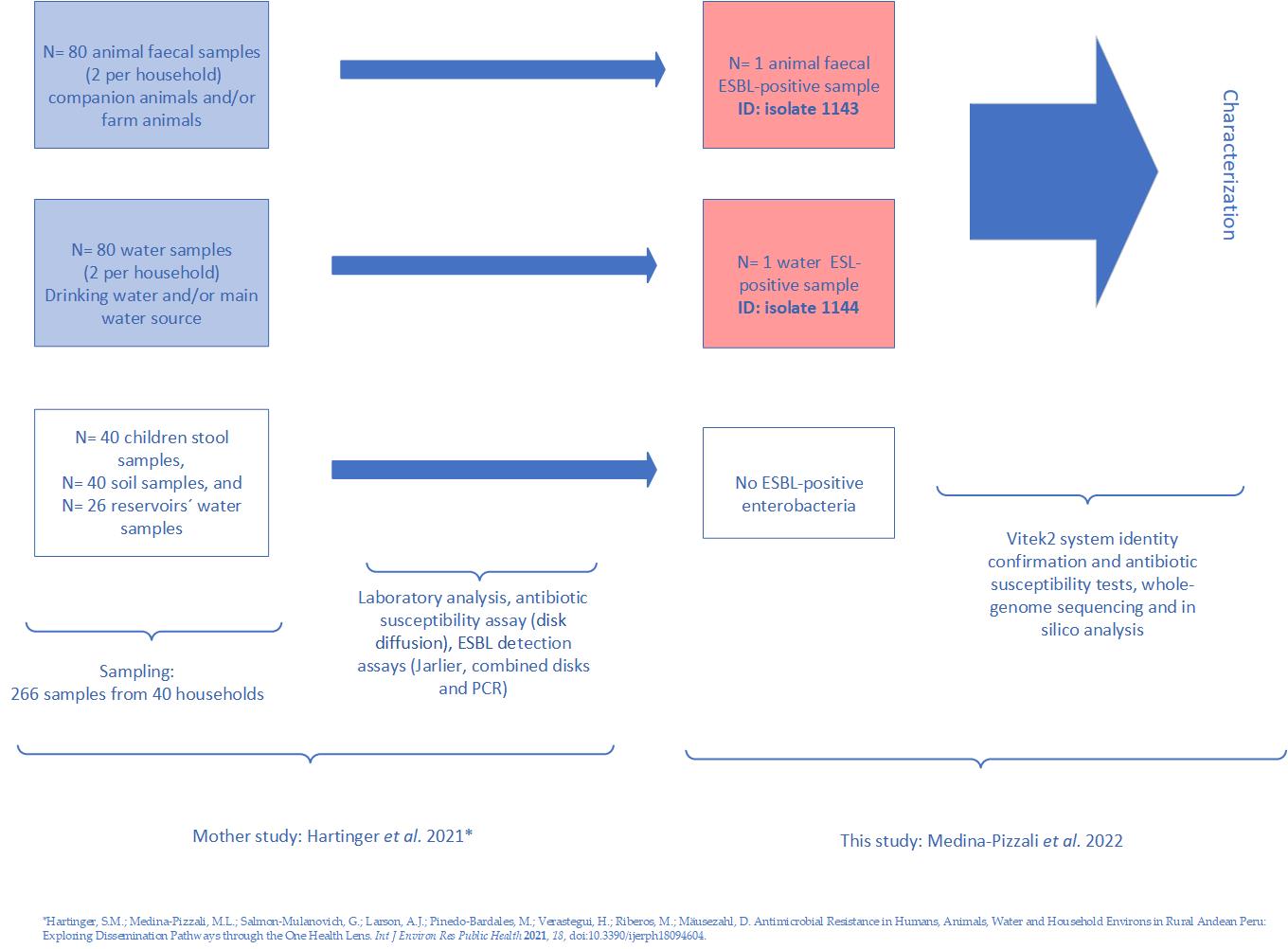

Supplement: Supplementary file 1 [file antibiotics-11-00692-s001.zip › Figure S1.jpg]
